# Supplementary material for: A Universal Fluorescent Immunochromatography Assay Based on Quantum Dot Nanoparticles for the Rapid Detection of Specific Antibodies against SARS-CoV-2 Nucleocapsid Protein
Source: Int J Mol Sci. 2022 Jun 2;23(11):6225. doi: 10.3390/ijms23116225 (PMC9180975; doi:10.3390/ijms23116225)
Supplement: Supplementary file 1 [file ijms-23-06225-s001.zip › ijms-1754731-supplementary.pdf]

Supplementary Material

Supplementary Figures and Tables

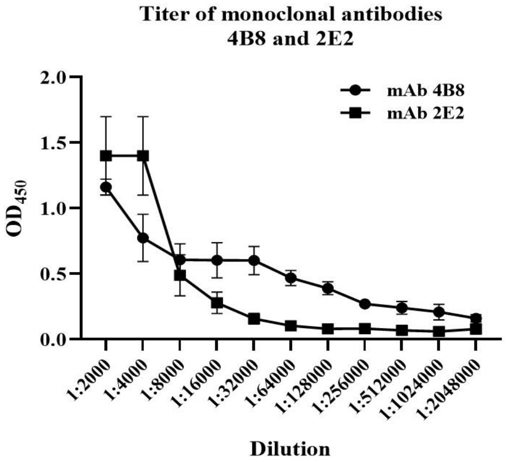

Supplementary Figure S1. Titer of monoclonal antibodies 4B8 and 2E2, tested by enzyme linked immunosorbent assay (ELISA).

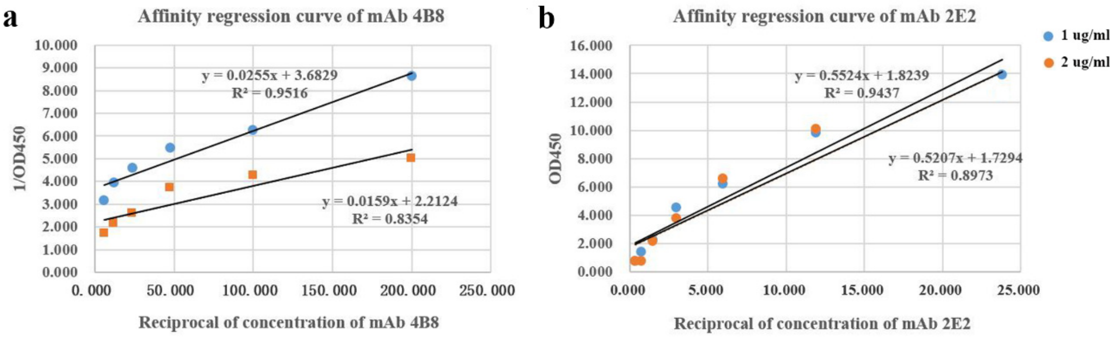

Supplementary Figure S2. The affinity regression curve of mAb 4B8 and 2E2 tested by enzyme linked immunosorbent assay (ELISA). The Kaff was calculated by the formula: Kaff=(n-1)/2(n[Ab']t-[Ab]t), n=[Ag]t/[Ag']t.

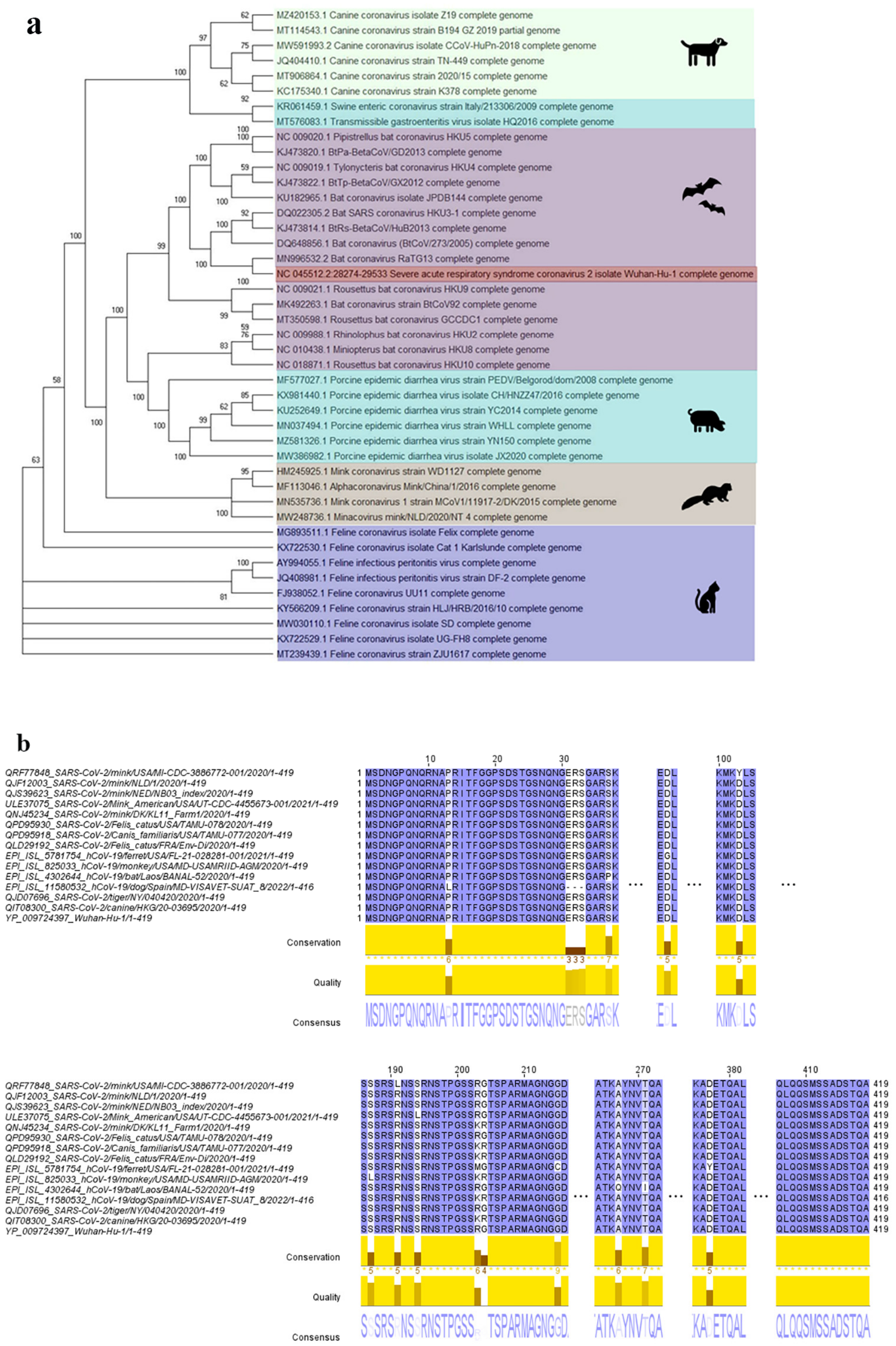

Supplementary Figure S3. Bioinformatical analysis. (a) Phylogenetic tree of coronaviruses of mammals (dog, bat, pig, mink and cat) which were homologous to SARS-CoV-2 in varying degrees. (b) Sequence alignment of 15 SARS-CoV-2 variants isolated from mammals.
